# Supplementary figures and images for: Gut bacteria influence Blastocystis sp. phenotypes and may trigger pathogenicity
Source: PLoS Negl Trop Dis. 2023 Mar 29;17(3):e0011170. doi: 10.1371/journal.pntd.0011170 (PMC10057785; doi:10.1371/journal.pntd.0011170)

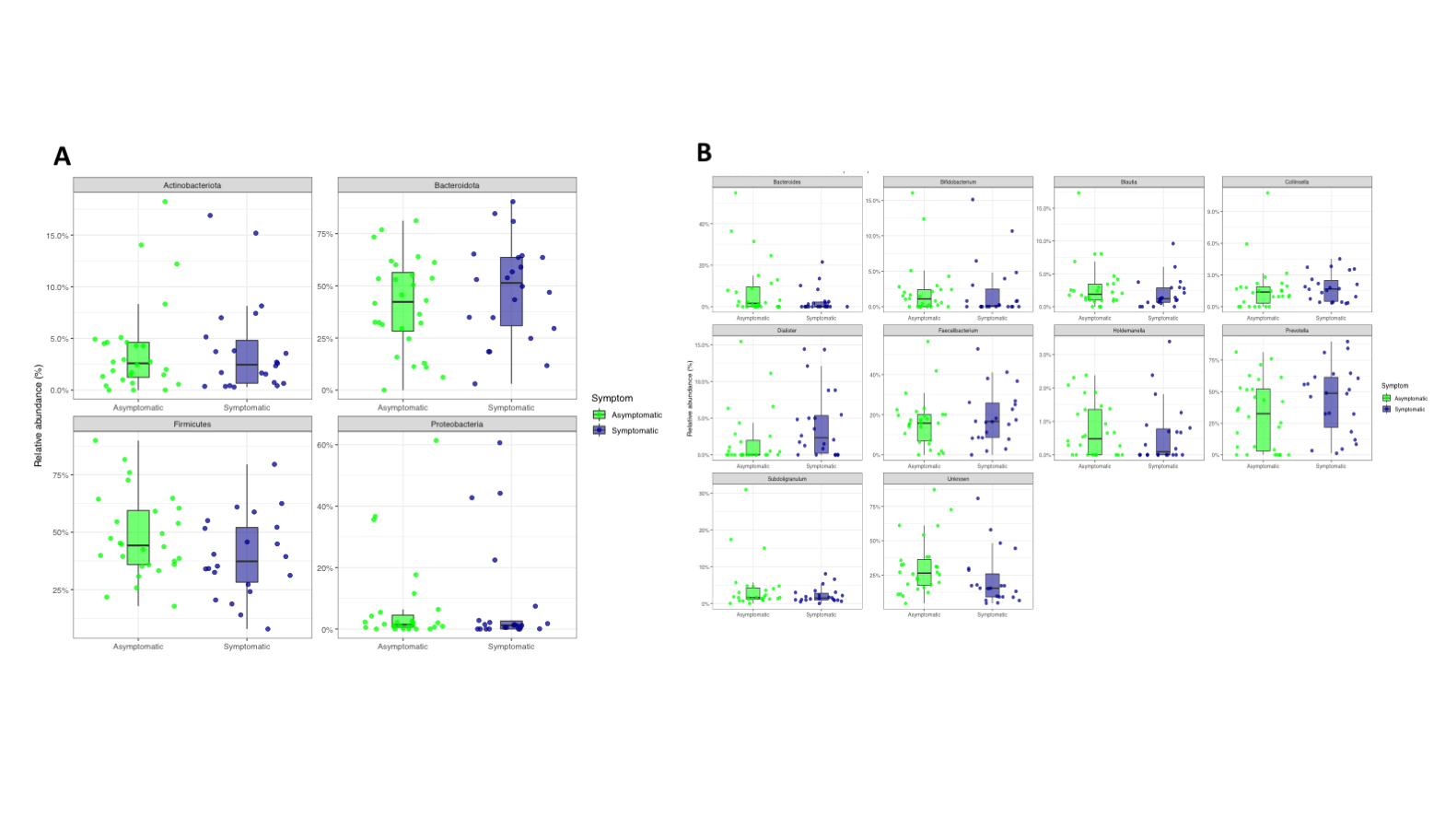

Supplement: S1 Fig — Breakdown of core microbiota at (A) phylum and (B) genus level in symptomatic and asymptomatic individuals. (TIFF) [file pntd.0011170.s002.tiff]

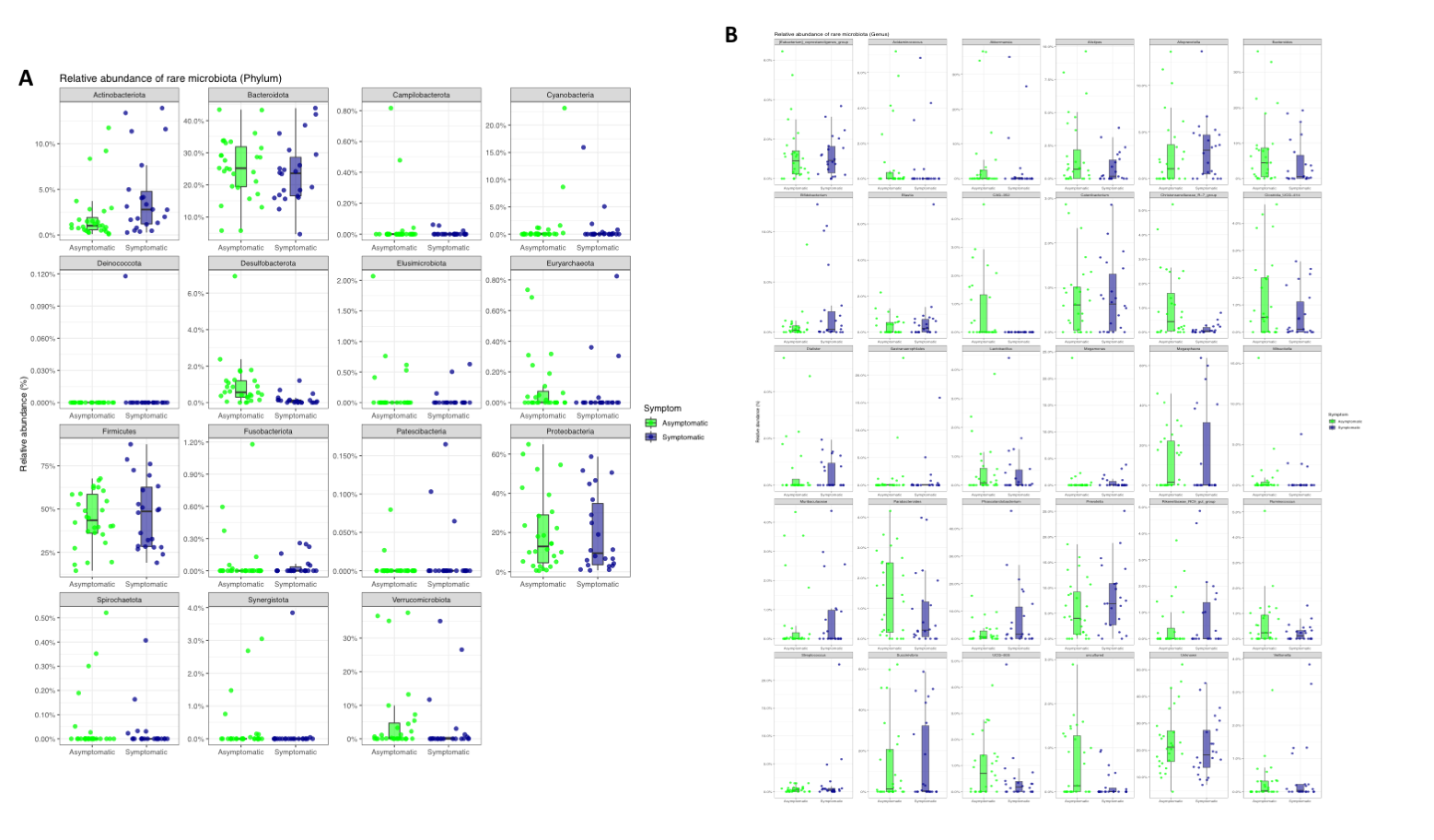

Supplement: S2 Fig — Breakdown of rare microbiota at (A) phylum and (B) genus level in symptomatic and asymptomatic individuals. (TIFF) [file pntd.0011170.s003.tiff]

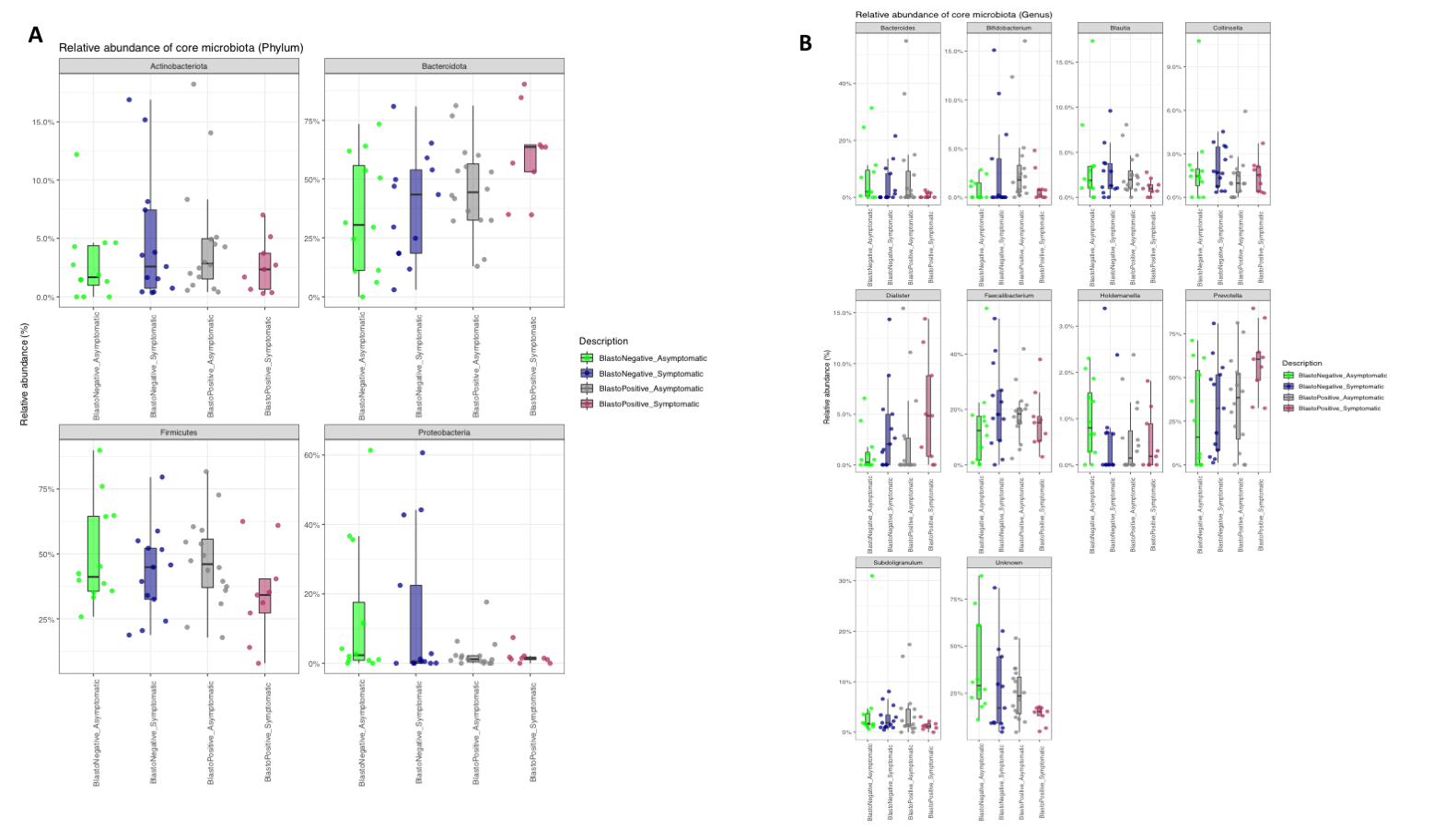

Supplement: S3 Fig — Breakdown of core microbiota at (A) phylum and (B) genus level in symptomatic and asymptomatic individuals with and without Blastocystis sp. colonization. (TIFF) [file pntd.0011170.s004.tiff]
