# Supplementary material for: Gut bacteria influence Blastocystis sp. phenotypes and may trigger pathogenicity
Source: PLoS Negl Trop Dis. 2023 Mar 29;17(3):e0011170. doi: 10.1371/journal.pntd.0011170 (PMC10057785; doi:10.1371/journal.pntd.0011170)
Supplement: Attachment — Submitted filename: Response to Reviewers.docx [file pntd.0011170.s005.docx]

5 October 2022

Editors-in-Chief

PLoS Neglected Tropical Disease

Dear Sir/Madam,

I am writing to notify you of the revised manuscript submission. We have appropriately addressed and carefully followed the reviewer's comments and suggestions.

Two manuscript file has been prepared according to the format set by PLoS. With this letter, I am also enclosing our responses to the reviewer's comments. Please note that some additional data requested by the reviewers have been submitted as supplementary figures and tables.

I hope the manuscript, which has highlighted an important aspect of *Blastocystis* sp. research will find its way towards acceptance.

Thank you for your consideration of this manuscript.

Sincerely,

**PROF. DR. SURESH KUMAR GOVIND**

Department of Parasitology

University of Malaya

Malaysia

**JABATAN PARASITOLOGI**

Fakulti Perubatan, Universiti Malaya, 50603 Kuala Lumpur, MALAYSIA

Tel: (603) 7967 4746 / 7967 4745 **•** Fax: (603) 7967 4754

**Response to Reviewer 1**

**Methods**

| **Num** | **Reviewers Comment** | **Response** |
| --- | --- | --- |
| 1. | Please present the demographic characterizations of both groups in a table | A supplementary table describing participants’ demography, underlying disease, and infection status have been included in the submission.  Please refer to Supplementary Table 1 |
| 2. | Please indicate whether healthy individual or infected groups had underlying diseases (e.g., diabetes, autoimmune disorders, etc.) or other gut infections | The exclusion criteria for this study were those with irritable bowel syndrome (IBS), inflammatory bowel disease (IBD), antibiotic consumption within the last month, and cancer. However, A supplementary table describing participants’ demography, underlying condition, and infection status has been included in the submission. |
| 3. | Please indicate B. hominis group had single infection and did not have co-infection with other gut pathogens or parasites. | All the participants who tested positive for *Blastocystis* sp. were also screened for other infections to ensure *Blastocystis* sp. is the sole infective agent. The manuscript is amended accordingly.  Please refer to lines 104-107. |
| 4 | Please add a statistical analysis section at the end of material and methods. | A section for statistical analysis was added.  Please refer to lines 257-262. |

**Results**

| **Num** | **Reviewers Comment** | **Response** |
| --- | --- | --- |
| 1. | Please add accession numbers if you deposited your sequences in GenBank | The accession number of the sequences was added to the manuscript.  Please refer to lines 267-269. |

**General comment**

| **Num** | **Reviewers Comment** | **Response** |
| --- | --- | --- |
| 1. | Please read and use the recent paper regarding asymptomatic B. hominis and gut bacteria composition: Behboud, et al. "Alteration of gut bacteria composition among individuals with asymptomatic Blastocystis infection: A case-control study." Microbial Pathogenesis (2022): 105639 | The recent paper has been added to increase justification on *Blastocystis* sp. infection in asymptomatic individuals.  Please refer to lines 446-448 |
| 2. | Please say your strengths, limitations, and suggestions for future researchers as a concise paragraph before conclusion section | The strengths, limitations and suggestions were added to the manuscript as suggested.  Please refer to lines 539-547 |

**Response to Reviewer 2**

**Methods**

| **Num** | **Reviewers Comment** | **Response** |
| --- | --- | --- |
| 1. | The authors need to articulate in the methods the definition what is meant by symptomatic and asymptomatic. Presumptively, this means intestinal symptoms (? diarrhea). Were these definitions made a prior or only after participant recruitment and questionnaire review. Given the potential range of 'subclinical' associations between a disease state and presence of Blastocystis described in the introduction it is critical that the report be specific about what disease condition is being studied. | *Blastocystis* sp. is often associated with non-specific gastrointestinal symptomatic apart from irritable bowel syndrome and colorectal cancer. In this study, the symptomatic patients are those individuals experiencing only non-specific gastrointestinal symptoms such as bloating, abdominal cramps, and diarrhea. While the asymptomatic individuals are those individuals who volunteer for stool screening during health camps and do not experience any gastrointestinal symptoms. The criteria were clearly defined even before the recruitment of patients. These individuals in the age group of 30-60 usually visit the gastroenterology specialist for consultation and screening. We have amended the manuscript with details to add more clarity.  Please refer to lines 95-102. |
| 2. | It appears that there were 50 individuals and each provided one sample. Please indicate this clearly. | The manuscript was amended as suggested.  Please refer to lines 94-95. |
| 3. | 'maintaining utmost anonymity' is vague. Please elaborate on why written consent was not required and the procedures for de-identification of samples | Our attempt to simplify and make the methodology concise left behind some essential details. We have, however, included these details to add clarity. Written consent was obtained from all individuals to show their willingness to participate in this study. However, the participants had requested that their personal details be kept anonymous. We have complied with that, especially in the demographic data of the population.  Please refer to lines 111-112 |
| 4. | The recruitment procedures and any inclusion/exclusion criteria are absent. Coupled with a lack of definition for symptomatic vs asymptomatic, it makes it difficult to appraise the assignment of different groups. | More clarity has been added to this in the methodology section.  Please refer to lines 108-112. |
| 5. | Please include a section for assignment of Blastocystis carriage status. What assignment based on culture recovery of parasites? qPCR data for Blastocystis would be more sensitive and allow for analyses based on parasite burden. | As suggested, a section for assignment of Blastocystis sp. carriage status has been included.  Please refer to line 93.  Those parasite cells isolated from symptomatic individuals are assigned as symptomatic isolate and vice versa.  This study assesses qualitatively the presence of *Blastocystis* sp. infection. Hence, *in vitro* cultivation was used to detect the presence of *Blastocystis* sp. infection. Our studies, even in the past, has always included this detection method and have been proven to be sensitive in detecting live parasite cells from stool samples. However, the use of qPCR to identify parasite burden and its interaction with the gut microbiota will be included in our future studies. |
| 6. | Line 187 implies that there was a screen for pathogens in the stool samples, yet a list of what pathogens and by what method is not provided. If Blastocystis is a common co-pathogen, this should be considered in the interpretation of the 16S sequencing data.  What is meant in line 188-189, eg. in Line 189, which groups? | Throughout this study, we have only used stool samples which had *Blastocystis* sp. as the sole parasitic infection. We have however screened for parasitic infection in *Blastocystis* sp.-free individuals using formal-ether concentration technique. These details have been included in the manuscript.  Please refer to lines 104-107  The fecal samples were grouped into symptomatic and asymptomatic groups. Both this groups were further broken-down to *Blastocystis* sp.-infected and *Blastocystis* sp.-free groups. We have used 5 isolates of *Blastocystis* sp. each from symptomatic and asymptomatic groups. Amendments were made in the manuscript for better clarity.  Please refer to lines 200-202 |
| 7. | Please clarify if antibiotics are present in the Jones media. Though a reference for the media is provided, given the emphasis of this paper on parasite-bacteria interactions it is important to explicitly indicate what if any antibiotics were present in the media. | The details added as suggested.  Please refer to lines 206-209 |
| 8. | Line 192. delete 'asymptomatic' as an adjective modifying Blastocystis, the parasite. This allows the authors to delete the paranethetical (ie. 3-days-old Blastocystis sp isolated from asymptomatic individuals...) | The word ‘asymptomatic’ deleted as suggested.  Please refer to the line 206. |

**Results**

| **Num** | **Reviewers Comment** | **Response** |
| --- | --- | --- |
| 1. | It is customary to show the letter designation for figure panels in the upper left, not the upper right. | The figures were amended accordingly. |
| 2. | If separating the symptomatic from asymptomatic, groups, then report the mean of all 50 participants and separately the mean in symptomatic vs asymptomatic for each group. Likewise, a breakdown comparison of phyla and genera between symptomatic and asymptomatic should be performed. | The mean value stated in Line 238 indicates the mean number of reads per sample generated from the NGS sequence analysis. Comparing this mean between symptomatic and asymptomatic does not have much implication. However, a thorough comparison of these groups has been done at the phyla and genera levels in terms of abundance of core and rare microbiota. The data has been included in supplementary figures.  Please refer to lines 264-271 |
| 3. | Figure 2: Please include a sentence about the 'n' of Blastocysts positive vs neg (A) and including by clinical status (B). C, What determined the circle sizes? Where are the individual samples? Is this absolute range of dots (where are the dots?), 95% CI?, some other measure of range? | Figure 2 has been amended as suggested. The sample size representing each group has been added and the explanation for the ellipses has been included to add clarity. The size of the ellipse is determined by a 95% confidence interval for the samples (represented by each point) distributed according to the distance matrix. Additional statistics have been added to further enhance the details of the figure.  Please refer to Figure 2. |
| 4. | Lines 318-324: please reference to a figure or data to substantiate the claims made in the text.  In addition, please comment on what methods were used to validate that the phenotypic differences between Blastocystis isolated from symptomatic versus asymptomatic participants were due to Blastocystis specifically and not the co-cultured bacteria in these xenic cultures.  Can the authors axenize Blastocystis? | The reference to figure has been added as suggested.  Please refer to lines 349-354.  This comment by the reviewer is exactly what our entire study addresses. In the past, *Blastocystis* sp. were thought to show phenotypic variation when isolated from different conditions. In this study, it is proven that phenotype of *Blastocystis* sp. is highly influenced by the microbial environment. The bacteria surrounding the parasite in *in vitro* cultures were maintained aseptically upon isolating from the stool sample of each participants. We assume these accompanying bacteria is autochthonous and when they are altered the parasite’s phenotype changes. This observation corroborates the role of bacteria in the infection of *Blastocystis* sp. The culture remained the same when there was no alteration of accompanying bacteria(Control experiments). This validates that phenotype seen in the parasite cells is due to the bacteria. However, the mode of interaction and the specific bacteria that trigger this is still unknown and are being studied now. Our study also shows that *Blastocystis* sp. infection alters the microbial composition as seen in other recent publications. It seems like there is a 2-way interaction which need further elucidation.  We have attempted to axenize *Blastocystis* sp. culture in a separate study but its characteristics gets altered too. Our research aims to retain the natural characteristics of the parasite by mimicking its natural environment. However, with the current findings suggesting pivotal role of bacteria, our future studies will include axenized *Blastocystis* sp. controls to study the specific interaction with different bacterial species. |
| 5. | Lines 327-334: does filtered or heat-killed bacterial suspension have a similar result? | Our study has used only bacterial suspension to elucidate the effect of live bacteria on *Blastocystis* sp. culture hence we have excluded the effect of filtered or heat-killed bacterial suspension. However, the effects that excretory and secretory products of bacteria may have on *Blastocystis* sp. is being currently studied. |
| 6. | Line 336: please indicate how the authors know that the protease activity is a function of Blastocystis rather than the bacteria present in symptomatic xenic cultures. protease activity could be a composite of Blastocystis and bacteria/other microbes present in the culture. | The method to study protease activity in *Blastocystis* sp. has been optimized in our laboratory and published previously:  Rajamanikam and Govind: Amoebic forms of *Blastocystis* spp. - evidence for a pathogenic role. Parasites & Vectors 2013 6:295.  Similarly, in the present study, we have used *Blastocystis* sp. isolates that have been purified to minimize bacterial contamination with a negligible bacterial protease activity. This ensures that the protease activity seen is predominantly by *Blastocystis* sp.  However, a statement is added in methodology section to add more clarity. Please refer to lines 235-236. |
| 7. | Line 348: How was the blastocysts antigen prepared and what measurements were done to confirm its purity compared with other proteins, including bacterial antigens, that may be present in the extract.  Also, numerically, the difference in proliferation between Blastocystis antigen from asymptomatic (20%) and symptomatic (60%) stool is similar (and with less variability) to asymptomatic (20%) and co-culture with bacteria (~65%). Please clarify. Also, both figures in 6B read 'bacterial suspension from asymptomatic parasite culture'. This seems inconsistent with the text.  Finally, is 'control' bacteria-free (axenic) or is 'control' with bacteria in the autochthonous xenic culture used to recover Blastocystis? | The solubilized antigens from *Blastocystis* sp. were extracted using the commonly accepted, freeze-thaw technique using liquid nitrogen. Prior to extraction of antigen, the *Blastocystis* sp. cells were purified to minimalize bacterial contamination. This ensures that, even the bacteria present will have negligible effect on the antigen. The antigens were measured using Bradford Protein Assay (BioRad). The *Blasatocystis* sp. antigens concentration was normalized to eliminate the effect of bacterial antigen.  The details have been added to the manuscript. Please refer to lines 237-239.  The asymptomatic isolates had a mean proliferation rate of 20% whereas the mean proliferation by symptomatic isolates is 60%. There was a slight and insignificant increase in the proliferation by symptomatic when cultured with asymptomatic bacterial suspension. The asymptomatic isolate’s proliferation rate increased from 19.8% to 65.8% when cultured with bacterial suspension symptomatic isolates. The confusion could have been due to the typographical error in the title of the plot in Figure 6B. The title have been amended accordingly.  The control experiments are parasite culture with autochthonous bacterial composition without any alteration to its bacterial composition. However, in the experiments, the control tubes received the same amount of dilution with sterilized Jones medium. This ensures a fair comparison.  Please refer to lines 221-223. |

**Discussion and Conclusion**

| **Num** | **Reviewers Comment** | **Response** |
| --- | --- | --- |
| 1. | That Blastocystis is accompanied by decreased alpha diversity and Pielou's evenness does not distinguish causality from association.  In addition to potential strain-dependent influences the authors reference in lines 380-385, there are potentially other factors (environmental, individual patient, population, and methodological) that could explain a discrepancy between findings in this study and other manuscripts.  Another interpretation is that the lower diversity stool selected for assignment of Blastocystis status (and therefore a molecular method to assign Blastocystis status should be pursued).  It is harder to culture Blastocystis ST3 from some stool (ie. symptomatic as shown in Figure 5) than other stool and perhaps not surprisingly stool with less microbial diversity is less likely to have bacteria that inhibit Blastocystis growth. | We agree with the reviewers comment that the decrease in alpha diversity not indicating causality. We have even acknowledged it in the discussion (Lines 524-526). However, we believe that understanding the correlation in *Blastocystis* sp. infection from different clinical status will be the way forward in understanding *Blastocystis* sp. completely.  We appreciate the reviewer’s comments. We have added the factors to the manuscript.  Please refer to lines 412-416.  This interpretation is highly likely but there isn’t enough evidence supporting this. Most studies currently focus on a single population. Also, there is no molecular method available currently to assign *Blastocystis* sp. status (Symptomatic or asymptomatic). The identification of *Blastocystis* sp.-specific factor would enable such method to be enabled.  In our experience, *Blastocystis* sp. from symptomatic individuals are not hard to culture but it has a characteristic growth profile that produces lower cell peaks. There could be bacteria that inhibits the growth of *Blastocystis* sp. which we are not able to rule out yet. Furthermore, amoebic forms and surface fuzzy coats, which enhances interaction and engulfment of bacteria have been observed in symptomatic isolates of *Blastocystis* sp. predominantly. This suggests the ability of symptomatic isolates to alter the microbial composition. There seems to be a 2 way interaction. The manuscript was edited to include these points in the discussion.  Please refer to lines 409-419. |
| 2. | The conclusion overstates what was observed. Line 506 extends beyond the data observed in this manuscript. There is no measure of immune barrier and immune response in this study and such a conclusion is only conjecture. Also, the designation of Blastocystis as either commensal or parasite based on the data presented is not possible. There is no evidence that a) Blastocystis is contributing to a disease condition in the participants or that b) the in vitro readouts are sufficient to designate a 'parasitic' phenotype. Rather than stating 'here we present', the authors could raise their new hypothesis that interactions between specific intestinal microbiota and Blastocystis influence whether the protozoan is functioning in a commensal or parasitic role.  Finally, Line 510, I think the authors would agree that the evidence presented is not strong. The study may be suggestive of the statement, but the presented data is not a complete mechanism | This statement was based on extrapolation of the current findings to imply the consequence of symptomatic *Blastocystis* sp. infection. However, we have removed the lines that are overstated in order to improvise the conclusion section.  Please refer to lines 550-558.  We agree to the reviewer’s comment. The findings from this study, although do not provide a strong evidence, we are convinced that there is a preliminary basis that sheds light on the controversial behavior of *Blastocystis* sp. as reported in various studies. We agree with the need for a more data and control experiments in providing a strong evidence for this claim. The conclusion section has been amended accordingly.  Please refer to lines 554-558 |

**Overall comments and additional specific comments**

| **Num** | **Reviewers Comment** | **Response** |
| --- | --- | --- |
| 1. | Overall the authors present an intriguing concept that microbiota-protozoa interactions are critical for understanding intestinal protozoan behavior (in this case Blastocystis) and disease potential. These are difficult hypotheses to test and prove, and the authors used an in vitro system of mixed stool culture derived Blastocystis with bacteria present in the same stool or from stool from participants with a different clinical status. The strength of the study is the attempt to disentangle these complex microbe-microbe interactions. Weaknesses, however, are several including: incomplete descriptions of the participant cohort (especially the definition of symptomatic or asymptomatic), apparent lack of a molecular assay to assign Blastocystis status (and therefore the designation of Blastocystis status is contingent upon culture viability which that authors demonstrate may be influenced by autochthonous bacteria in the stool), apparent lack of axenic Blastocystis controls, and lack of reporting what measurements or manipulations were performed to indicate that the phenotypic changes seen from different mixes of microbial communities were derived from changes in Blastocystis rather than derived from the other microbes in the xenic assay system. Additional experiments are therefore needed to reach the authors' final conclusions. | The weaknesses highlighted by the reviewer have been mostly addressed appropriately.   1. *incomplete descriptions of the participant cohort (especially the definition of symptomatic or asymptomatic)*   The description has been added in detail and demographic details of participants have been included.   2. *apparent lack of a molecular assay to assign Blastocystis status (and therefore the designation of Blastocystis status is contingent upon culture viability which that authors demonstrate may be influenced by autochthonous bacteria in the stool)*   To date, there are no molecular assays to determine *Blastocystis* sp. status. Our research team has consistently seen and published observations on phenotypic characteristics which are distinct among the different sources of isolations which validates the use of phenotypic characteristics to differentiate different groups and *Blastocystis* sp. status. Below are the references of studies that have been published previously and widely accepted:-   i. Chye, T. T. and K. G. Suresh (2006). "Predominance of amoeboid forms of *Blastocystis hominis* in isolates from symptomatic patients." Parasitology research **98**(3): 189-193. ii. Tan, T. C., et al. (2008). "Phenotypic and genotypic characterisation of *Blastocystis hominis* isolates implicates subtype 3 as a subtype with pathogenic potential." Parasitology research **104**(1): 85-93. iii. Ragavan, N. D., et al. (2014). "Phenotypic variation in *Blastocystis* sp. ST3." Parasites & vectors **7**(1): 404. iv. Rajamanikam, A. and S. K. Govind (2013). "Amoebic forms of Blastocystis spp. - evidence for a pathogenic role." Parasites & vectors **6**(1): 295. v. Rajamanikam, A., et al. (2022). "Distinct Phenotypic Variation of Blastocystis sp. ST3 from Urban and Orang Asli Population—An Influential Consideration during Sample Collection in Surveys." Biology **11**(8): 1211. vi. Franklin, F., et al. (2022). "Higher amoebic and metronidazole resistant forms of Blastocystis sp. seen in schizophrenic patients." Parasites & vectors **15**(1): 1-16. 3. *apparent lack of axenic Blastocystis controls*   Our study aims to alter the accompanying microbiota of the protozoa to study the alterations in its phenotype. Hence, we have attempted to retain the natural conditions of the protozoa in this preliminary assessment. We believe axenic controls will be better in use when the influence of a specific bacteria is assessed. This study is the first to investigate the role of accompanying bacteria in *Blastocystis* sp. However, in the studies involving antigens of the protozoan parasite, we have purified the cells to minimalize the contamination of bacteria to a negligible level resembling an axenized status.   4. *lack of reporting what measurements or manipulations were performed to indicate that the phenotypic changes seen from different mixes of microbial communities were derived from changes in Blastocystis rather than derived from the other microbes in the xenic assay system*   The stool samples selected for inclusion into the study was screened so that *Blastocystis* sp. was the sole infective agent. Hence, this ensures that the alterations in microbial communities are due to *Blastocystis* sp. colonization. |
| 2. | Abstract: multiple changes are necessary 'microbiome' to 'intestinal microbiota'. Reserve the use of the term 'microbiome' in reference to descriptions of the intestinal microbial community genomic structure. | The abstract section has been altered accordingly. |
| 3. | "healthy" could easily be a misclassification of individual status and should be avoided. Rather these 'healthy' individuals are Blastocystis ST3 negative controls. | The word “healthy” is changed to “*Blastocystis* sp.-free” as suggested.  Please refer to the line 35. |
| 4. | change line 44. This reads as an overstatement and 'symptomatic' or 'asymptomatic' refer to host syndromes, not characteristics of a parasite. It would be better to just state the observation without assigning presumed consequences on the host:eg. ".... demonstrated diminished growth and diminished expression of putative virulence genes".. | The statement was amended accordingly.  Please refer to lines 43-46 |
| 5. | line 47 'microbial diversity' appears to refer to the genomic (16S amplicon?) diversity. This should be more clearly stated. | The word ‘microbial diversity’ refers to diversity of microbial composition. We have amended the statement accordingly.  Please refer to the line 48 |
| 6. | The introductory paragraph is difficult to read, in part because of the sentence structure, and in part because of the complexity of the concepts. First, there are plenty of studies to indicate that plasticity in response to dietary and environmental influences is a fundamental characteristic of intestinal microbiota (and therefore not unexpected). The first sentence also inappropriately lumps direct influences with established influences on intestinal microbial community structure (ie. diet) with those that are indirect, or require several steps to prove causality (ie. climate change, to this reviewer's knowledge had not been proven to directly alter intestinal microbiota). It is important when introducing this concept that the authors take care to delineate what is known and verifiable from what is speculation (even if plausible). In Line 60 and line 62 it is also not clear what specific influences on the host are relevant for the submitted manuscript. Susceptibility to what? It would be better for this paragraph to do more to introduce the 'holes' in our understanding of the intestinal microbiota as not only prokaryotes but also eukaryotes (as introduced in lines 66-67). | The paragraphs of the introduction section has been streamlined to be more specific.  Please refer to lines 56-61 |
| 7. | Line 76 would be more accurate to state "increased diversity of bacteria in the presence of intestinal Blastocystis carriage." The prior sentences appropriately explain the questionable pathogenicity of this parasite, so the designation of 'infected gut' is therefore controversial. | The statement have been changed to “Studies have reported on increased diversity of bacteria in *Blastocystis* sp. colonized gut”.  Please refer to lines 70-71. |
| 8. | Please do not use the terms 'flora' and 'microbiota' and 'microbiome'. Suggest changing 'flora' to 'microbiota' for consistency. Otherwise it is not clear what the difference is between flora and microbiota and whether these terms are synonymous or meant to designate something different to the reader. | The manuscript have been amended to streamline the terminology used. |
| 9. | The references to Giardia in line 86 are too restricted. Please include references to other studies including Bartelt et al PLoSPathogens, 2017; work by Andre Buret laboratory and work by Scott Dawson/Steven Singer group. For E histolytica see papers by Stacey Burgess. | The suggested references were included accordingly.  Please refer to lines 80-82 |
