# Supplementary material for: Gut bacteria influence Blastocystis sp. phenotypes and may trigger pathogenicity
Source: PLoS Negl Trop Dis. 2023 Mar 29;17(3):e0011170. doi: 10.1371/journal.pntd.0011170 (PMC10057785; doi:10.1371/journal.pntd.0011170)
Supplement: Attachment — Submitted filename: Response to Reviewer 2.docx [file pntd.0011170.s006.docx]

**Response to Reviewer 2**

**Methods**

| **No.** | **Reviewers comments** | **Response** |
| --- | --- | --- |
| 1. | Important clarifications have been made. There are still some apparent inconsistencies to resolve, for example "control" in Figure 6A and 6B don't appear to refer to the same thing, and that is confusing and requires significant work by the reader to comprehend (these concepts are complex) | The control in both Figure 6A and 6B refers to the same thing. There was an error in the labelling earlier which was amended now. The label ‘Bacteria Co-cultured’ had caused a confusion. The label was changed accordingly. There are two experimental groups – control and co-cultured. Control refers to the xenic culture that only received sterile Jones medium whereas co-cultured refers to the xenic cultures that received bacterial suspension of symptomatic or asymptomatic cultures. The control has been defined in lines 224-227. The definition was also added in the legend of Fig 6. |

**Results**

| **No.** | **Reviewers comments** | **Response** |
| --- | --- | --- |
| 2. | Regarding comment #4/6. To clarify, these questions addressed how the authors knew that the protease activity (figure 6A) was due to Blastocystis proteases and not bacterial-derived proteases that are different between different bacterial communities. Part of the confusion for this reviewer was that the terms "control" and "bacterial co-culture" in Figure 6 are not well defined elsewhere in the manuscripts. Based on the methods (new line 239), it is now clearer that 'control' means 'minimalized bacteria' where as 'bacterial co-culture' means the the non-bacterial minimalzied/standard xenic culture. From a different perspective, the experimental group is therefore the minimalized bacterial group if blastocysts culture normally includes the autochthonous xenic bacteria (conversely, in Figure 6B it appears that control indeed does refer to autochthonous bacterial culture). Line 239 helps to alleviate concerns that the bacterial proteases account for differences in Figure 6A. I'd suggest re-labeling Figure 6A as following "Bacterial co-cultured" should read "autochthonous culture" and "control" should read "minimalized bacteria culture". This makes more clear what was done and what is being compared. | At the end of experiments involving alteration of surrounding microbiota*,* the *Blastocystis* sp. cells from co-cultured and control experiments were purified to minimalize bacterial contaminations. The minimal bacteria contaminant was tested to have negligible protease activity. This confirms that the protease activity found in the solubilized antigens belonged to *Blastocystis* sp. The definition of control and co-cultured were clearly defined in lines 224-227, legend of Fig 6 and other parts of the methodology(Highlighted in the manuscript).  The control experiment is actually *Blastocystis* sp. with its autochthonous bacteria as there was only Jones medium added. While in the co-cultured experiments, bacterial suspension from symptomatic or asymptomatic were added. The comparison was made between control and co-cultured to understand the influence of changing surrounding bacteria of *Blastocystis* sp. Hence, we think that the relabeling, as suggested by the reviewer is not necessary since sufficient clarification have been added to the manuscript to address the inconsistencies highlighted by the reviewer. |
| 3. | Line 383: This paragraph needs some editing for grammar and clarity. It is still hard to tell what is going on. First, include the term colonic cell proliferation so it is clear what is being measured. For example, change the opening clause to read "Regarding the ability of Blastocystis antigens to promote colonic cell proliferation,..." instead of "When tested on the ability to proliferate cells". | The manuscript is amended accordingly.  Please refer to line 385. |
| 4. | Line 384, should 'individual' be 'individuals'? Also, should the comma be removed? The authors are meaning antigens from Blastocystis isolated from symptomatic individuals that were later cultured with the bacteria from asymptomatic individuals. Correct? | The amendments were made accordingly.  Please refer to lines 386-388 |
| 5. | Line 384, 'bacteria from asymptomatic INDIVIDUALS' not bacteria from asymptomatic isolates | The manuscript was edited accordingly.  Please refer to lines 386-388 |
| 6. | Line 385-386 should this read "Nonetheless, [?ANTIGENS from?] Blastocystis sp isolated from asymptomatic individuals that were cultured with bacteria from symptomatic individuals produced greater proliferation than antigens from Blastocysits isolated from asymptomatic individuals in autochonthonus culture?" As written in line 387 it isn't explicit what comparison was made. Greater than what? | The amendments were made accordingly.  Please refer to lines 388-391 |

**Conclusions**

| **No.** | **Reviewers comments** | **Response** |
| --- | --- | --- |
| 7. | The toned down conclusion is much better and appropriate to the data presented.  However, new paragraph, Lines 542-550 needs revision. In Line 545-546, again the findings are associative and not causal. Instead of 'due to' the authors should state "associated with" ... a single subtype.  Also, the only other pathogens screened were other parasites (not other bacteria or viruses) and the parasite diagnostics used were low sensitivity non-molecular based methods. So it is overstating to say that Blastocystis was a single infective agent. The entire clause "which is also a single infective agent" should be removed.  Please add the limitation of not use molecular diagnostics and that therefore a) Blastocystis-free group may have had individuals with Blastocystis that wasn't cultivatable by the authors methods and that b) Blastocystis quantitative burden in stool cannot be compared between the two symptomatic groups. | The amendments were made and suggestion were incorporated in the manuscript.  Please refer to line 554.  The sentence was removed as suggested. Please refer to line 554.  The limitation was added.  Please refer to lines 549-553. |

**Summary and General Comments**

| **No.** | **Reviewers comments** | **Response** |
| --- | --- | --- |
| 8. | The authors have provided a detailed response to most of the critiques. Some responses were misinterpreted, but they are minor and need not be further addressed. (eg. by Blastocystis status I did not mean differentiating symptomatic from asymptomatic, but rather presence or absence of Blastocystis. In this paper they used culture-recovery methods rather than molecular methods to assign presence or absence of Blastocystis. Future studies should incorporate qPCR if possible--https://journals.asm.org/doi/10.1128/JCM.01392-10). Overall I think the findings are of interest, appropriate for PLoSNTDs, but a few more revisions are needed before this is publication-ready. | The authors appreciate the reviewer’s suggestion. qPCR will be incorporated in future studies. |
| 9. | In addition to specific section comments above, please pay careful attention to semantics in the paper. As in line 209, Line 217 should read "Three-day-old Blastocystis sp. cells from symptomatic individuals". The Blastocystis cells are not inherently symptomatic or asymptomatic.  This same problem is present in Figure 6 legend where there is reference to 'symptomatic' and "asymptomatic" parasite culture.  It happens again in lines 357-359 where line 357 reads "symptomatic individuals' whereas line 358 reads "asymptomatic isolates". Line 376 should read "Isolates from asymptomatic individuals".  Line 378 should read "bacterial suspension from symptomatic individuals". Please correct throughout the manuscript. For this reason I recommended additional copyediting below. | Corrected.  Please refer to line 217.  Corrected.  Please refer to legend of Fig 6.  Corrected.  Please refer to lines 358-361  Corrected.  Please refer to line 380 |
| 10. | Finally, and now that clarifications have allowed me to better comprehend this study, I suggest a change in the title. The authors don't show 'harm' from Blastocystis in the in vitro models and likewise it is not possible from the data presented to assign the organism as 'harmless'. A more appropriate title would be "Gut bacteria influence Blastocystis phenotypes and may trigger pathogenicity" | The title was amended as suggested. |
